# 2464: Phenotypic characteristics of pediatric nonalcoholic fatty liver disease

**DOI:** 10.1017/cts.2017.111

**Published:** 2018-05-10

**Authors:** Shima Dowla, Ambika Ashraf, Stella Aslibekyan

## Abstract

OBJECTIVES/SPECIFIC AIMS: The purpose of this study is to characterize children with nonalcoholic fatty liver disease (NAFLD) living in the Southeastern United States. METHODS/STUDY POPULATION: This retrospective electronic medical record chart review was conducted on a random sample of 206 children identified with NAFLD. Patients were included if they met the following criteria: confirmed NAFLD through either an ultrasound or liver biopsy or clinical suspicion of fatty liver disease alongside elevated alanine aminotransferase (ALT) in the absence of other etiologies causing elevated transaminases. Patients were excluded if they had hepatitis or other documented liver disease. Data collected at initial presentation included age, gender, ethnicity, height, weight, body mass index (BMI), BMI percentile, blood pressure, HbA1c, aspartate aminotransferase (AST), ALT, γ-glutamyl transferase (GGT), total cholesterol, total triglycerides, low-density lipoprotein, and high-density lipoprotein. Statistical analysis: for descriptive statistics, frequency counts and percentages alongside means, standard deviation, range, min/max values for the continuous variables were calculated. RESULTS/ANTICIPATED RESULTS: This study included 206 children diagnosed with NAFLD. Subjects were primarily male (n=136, 66%) and Caucasian (n=133, 66%), followed by Hispanic (n=42, 21%), Black (n=25, 12%), and Asian (n=2, 1%). Mean age at diagnosis was 12.3±3.5 years. Mean weight (lbs), height (in), and BMI (kg/m^2^) of subjects at diagnosis were 192±77 lbs, 61.7±6.6 in, 34.6±9.7 kg/m^2^, respectively. Patients had an average systolic blood pressure of 124±15.4 mmHg and diastolic blood pressure of 69.6±10.6 mmHg. Mean ALT was 91.8±67.2 U/L, AST was 61±38.8 U/L, and GGT was 55.1±64.6 U/L. Mean HbA1c was 5.8±1.4%, cholesterol was 176±36.3 mg/dL, triglycerides were 200±134 mg/dL, low-density lipoprotein was 107.6±32.1 mg/dL, and high-density lipoprotein was 39.9±8.4 mg/dL. DISCUSSION/SIGNIFICANCE OF IMPACT: In addition to having significantly elevated liver enzymes, children with NAFLD had several derangements in their metabolic profile, most notably high triglyceride levels and HbA1c values in the prediabetic range. Although lifestyle modification is the gold standard treatment for NAFLD, pharmacotherapy may need to be included to address metabolic syndrome.

